# Kidney function and use of nonsteroidal anti-inflammatory drugs among elderly people: a cross-sectional study on potential hazards for an at risk population

**DOI:** 10.1007/s11096-018-0598-8

**Published:** 2018-02-19

**Authors:** Sara Modig, Sölve Elmståhl

**Affiliations:** 1 Department of Clinical Sciences in Malmö, Division of Family Medicine, Lund University, Malmö, Sweden; 2 Department of Clinical Sciences in Malmö, Division of Geriatic Medicine, Lund University, Malmö, Sweden

**Keywords:** Anti-inflammatory agents, Elderly, Glomerular filtration rate, NSAID, Risk, Sweden

## Abstract

*Background* Renal elimination normally decreases with age. Nonsteroidal antiinflammatory drugs (NSAIDs) carry a risk of additional kidney damage. *Objective* The aims of this study were to assess the prevalence of NSAIDs in the elderly (aged ≥ 65) population in Sweden, explore reasons for any possible differences in the level of use and assess their kidney functions. *Setting* Data were obtained from the cohort study Good Aging in Skåne, Sweden. Patients aged 65 or more were included. *Methods* Medication lists were collected as well as variables such as cognition and education levels. Glomerular filtration rate was estimated from creatinine and cystatin C. Descriptive statistics and multiple linear regression analysis were used. Main outcome measure: NSAID use among the general elderly population. *Results* A total of 1798 patients were included. Approximately six percent (n = 105) of the people in the study group were using NSAIDs and of those 82 (78%) bought NSAIDs over the counter (OTC). 42% of those buying NSAIDs OTC showed an estimated glomerular filtration rate below 60 ml/min/1.73 m^2^. Education level did not affect the use of nonsteroidal anti-inflammatory drugs, nor did age. NSAIDs were more commonly used than other recommended analgesics. *Conclusion* Many people are unaware of the risks associated with the use of NSAIDs. The findings imply that the frailest elderly use NSAIDs to the same extent as the younger elderly do. It is important that information about safety of these drugs be communicated to both patients and healthcare professionals.

## Impacts on Practice

- The findings confirm the importance of medication reconciliation, and this must include questions about the use of over-the-counter medications.
- There is a need for information directed towards the general elderly population, since the findings suggest that many older people are unaware of the risks associated with non-steroidal antiinflammatory drugs, including risk of renal damage.
- It is essential to plan measures that ensure appropriate pharmacist counselling regarding non-steroidal antiinflammatory drugs.
- The communication between prescribers and pharmacists at community pharmacies is important.

## Introduction

Renal elimination normally decreases with age due to reduction in both renal blood flow and glomerular filtration (GFR) [1, 2]. In addition, many common conditions among the elderly, such as hypertension, diabetes and atherosclerosis, contribute to further reduction of the renal function. Renal elimination is the most important pharmacokinetic change that occurs in the elderly [3]. Among the population from the Good Aging in Skåne (GÅS) study, which is representative of the Swedish general population, a significantly decreased renal function after age 80 was demonstrated. More than 25% of the oldest demonstrated eGFR below 30 ml/min/1.73 m^2^, i.e. established renal failure of chronic kidney disease (CKD) grade 4 [2, 4]. Among Swedish nursing home patients, more than half of the population was found to have a renal failure of CKD grade 3 or more (< 60 ml/min/1.73 m^2^) [5]. In an elderly population with multiple illnesses from Iceland the corresponding proportion was over 70% [6].

Many medications are eliminated via the kidneys. Dose adjustment is therefore often necessary in elderly patients in order to avoid adverse effects and/or further impairment of renal function [3]. Some drugs are even contraindicated for patients with renal impairment. NSAIDs (nonsteroidal anti-inflammatory drugs) could be useful for musculoskeletal pain, but are potentially harmful for many elderly people due to the risk of kidney failure, heart disease or gastrointestinal bleeding [7–9]. NSAIDs carry a risk of gradual decrease of GFR as well as acute kidney damage [10–13], and NSAID users without previous renal disorder had a three-fold greater risk for developing acute renal failure compared with non-NSAID users in the general population in United Kingdom [14]. The Swedish recommendations state paracetamol as the number one analgesic for elderly people, with opioids, such as morphine, as the next step for stronger pain, despite the fact that the latter are associated with adverse effects, including sedation, dizziness and even confusion. Opioids should therefore only be used after strict consideration. These recommendations differ slightly from the WHO pain ladder, where NSAIDs are included in the first step and mild opioids, such as codeine, are recommended as a second step, before strong opioids [15].

The Beers Criteria includes avoidance of NSAIDs in CKD stage 4 and 5 [16] and the STOPP/START criteria considers the cut-off for use to be < 50 ml/min/1.73 m^2^ [17]. Nevertheless, the use of NSAIDs among the elderly was hazardously high in previous studies. Three out of four elderly patients discharged from hospital in the Netherlands after gastrointestinal bleeding were prescribed NSAIDs again within 7 years [18]. In Italy, 20% of the general population aged > 65 used NSAIDs in a typical week and 5.3% were chronic users [19]. In the nursing home population mentioned above, however, the use of NSAIDs was low, indicating knowledge exists about the risks among the Swedish prescribers [5]. These patients seldom obtain medications over the counter (OTC) themselves. However, in northern Sweden inappropriate prescriptions on the basis of impaired renal function were more common among patients with dementia living in nursing homes than among those living at home [20]. An educational intervention effectively reduced NSAIDs use in nursing homes in the US, where prescription of NSAIDs for these patients was more frequent [21].

Many NSAIDs are sold OTC in Sweden and the general population is often exposed to commercial advertising regarding these drugs. Other European citizens buy NSAIDs over the counter as well [19]. However, it is not common knowledge that these drugs are potentially harmful for a great proportion of the elderly population. Furthermore, many elderly people are not aware of their decreased kidney function.

## Aim of the study

The aim of this study was to assess how many in the general elderly population in Scania in Southern Sweden use NSAIDs regularly or as needed; what level of kidney function these people have and if there are any variables associated with possible differences in NSAID use.

## Ethics approval

The study was approved by the regional ethics committee at Lund University (LU 744-00). Written informed consent was obtained from all individual participants included in the study. The study has been performed in accordance with the ethical standards as laid down in the 1964 Declaration of Helsinki.

## Methods

### Setting

The study population was obtained from the ongoing Good Aging in Skåne (GÅS) study, part of the Swedish National Study on Aging and Care (SNAC), which has been described elsewhere in detail [22, 23]. This is a cohort study representative of the Swedish general population. The original GÅS population includes 2931 participants aged 60–93 years old from nine age cohorts. The participants live in five municipalities of different sizes and are randomized from the municipality registers with a participation rate of 60%.

Patients aged 65 or more were included in the present study. The study was designed as a cross-sectional study of 1832 subjects attending the 6-year re-examination year 2007–2011 out of 2264 survivors from the baseline (follow up rate 81%).

### Procedure

Information on the study population was collected on the basis of medical health examinations, psychological examinations, function tests and survey questions. In order to assess differences between groups regarding NSAID use, the following variables were used: education level, sex, age, place of living (city/countryside, i.e. access to care) and score level of mini-mental test (MMSE). Investigations were performed either at the research center or in the patient’s own home or at nursing homes to avoid selection bias.

Medication data consisted of stated intake from prescriptions as well as from OTC drugs and was collected by obtaining the patients’ medication lists, self-reported and/or written. The medication lists contained information about drugs used regularly as well as those used “as needed”. Access was given to medical records in order to check medications that were prescribed. It should be emphasized that the self-reporting procedure confers a greater certainty about which drugs were actually used out of those stated as “as needed”. The use of systemic NSAIDs was noted, as well as use of other analgesics, as a marker of chronic pain condition. In acute pain, systemic or local adverse event rates with topical NSAIDs were no greater than with topical placebo, and also in chronic pain, serious adverse events were rare [24]. The active substances (ATC groups) that were included are presented in Table 1, as are also the systemic NSAIDs that are available OTC in Sweden.

**Table 1:** Active substances for systemic use (ATC groups), that were included in the analysis

NSAIDs	M01AB01	Indometacin
	M01AB05	Diclofenac^b^
	M01AB15	Ketorolac
	M01AB55	Diclofenac, combinations
	M01AC01	Piroxicam
	M01AC02	Tenoxicam
	M01AC05	Lornoxicam
	M01AC06	Meloxicam
	M01AE01	Ibuprofen^a^
	M01AE02	Naproxen^a^
	M01AE03	Ketoprofen
	M01AE14	Dexibuprofen
	M01AE17	Dexketoprofen
	M01AE51	Ibuprofen, combinations
	M01AE52	Naproxen/esomeprazole
	M01AH01	Celecoxib
	M01AH04	Parecoxib
	M01AH05	Etoricoxib
	M01AX01	Nabumetone
Other analgesics		
Paracetamol	N02BE01	Paracetamol^a^
Opioids	N02AA01	Morphine
	N02AA03	Hydromorphone
	N02AA05	Oxicodone
	N02AA55	Oxicodone, combinations
	N02AA59	Codeine, combinations
	N02AB01	Ketobemidone
	N02AB03	Fentanyl
	N02AE01	Buprenorphine
	N02AG01	Morphine and spasmolytica
	N02AG02	Ketobemidone and spasmolytica
	N02AX02	Tramadol
	N02AX06	Tapentadol
Drugs for neuropathic pain	N03AX12	Gabapentin
	N06AA09	Amitriptyline
	N06AX21	Duloxetine

^a^Drugs for systemic use available OTC outside pharmacies (for paracetamol only other formulations than tablet since 2015)

^b^Drugs for systemic use available without prescription, though only at pharmacies

#### Laboratory measurements

Plasma creatinine (p-Cr) and cystatin C (p-cysC) were studied for the whole GÅS cohort [4]. P-cysC was, as one batch, measured by a fully automated particle-enhanced immunoturbidimetric assay [25] at Lund University Hospital. Normal reference range of p-cysC is specified as 0.63–1.44 mg/l (> 50 years) [26].

P-Cr was measured using a creatininase-based procedure on the Hitachi Modular P analysis system at Lund University Hospital. The method is calibrated to IDMS levels. Normal reference range of p-Cr is specified as 60–100 µmol/l for men and 50–90 µmol/l for women.

#### Estimated GFR

The levels of p-cysC and p-Cr were used as markers for glomerular filtration rate (GFR). Estimated GFR (eGFR) was calculated with the cystatin C-based CAPA [27] and the creatinine-based LM-Rev (Revised Lund-Malmö Study equation without body weight measure)—equations [28], as commonly used in Scania, Sweden. The mean of the two estimated GFR-values was used, as recommended as the generally best estimate for adults [29–31].

- *CAPA* : eGFR = 130 × cystatin C^−1.069^ × age^−0.117^ − 7,

- *LM Rev*; *pCr in* μmol/L
- e^X − 0.0158 × age + 0.438 × ln(age)^
- Female and pCr < 150: X = 2.50 + 0.0121 × (150 − pCr)
- Female and pCr ≥ 150: X = 2.50 − 0.926 × ln(pCr/150)
- Male and pCr < 180: X = 2.56 + 0.00968 × (180 − pCr)
- Male and pCr ≥ 180: X = 2.56 − 0.926 × ln(pCr/180)

Estimated GFR was categorized in CKD stages according to Kidney Disease Outcomes Quality Initiative guidelines (< 30, 30–59, ≥ 60 ml/min/1.73 m^2^) [32].

### Analysis

Descriptive statistics (frequencies, mean values and proportions) were used to describe the use of NSAIDs and the kidney function in the population.

Multiple linear regression analysis was performed with the use of NSAIDs as a dependent variable to assess if there were any explanation models for possible differences in NSAID use. The following independent variables were chosen: sex (male = 0, female = 1), age, education level (elementary school not completed/elementary school/secondary school/one or more year extra or university degree), place of living (urban and rural settings as a proxy for access to in-patient and outpatient) and score level of mini-mental test (MMSE). Two analyses were performed, one with NSAID use dichotomized as Yes/No and one with NSAID use split into No use/OTC use/NSAID prescribed, respectively.

The independent variables were checked for multicollinearity with Tolerance and Variation Inflation factors (VIF). No signs of multicollinearity were detected.

Analyses were performed in IBM Corp. SPSS Statistics for Windows, Version 22.0, Armonk, NY, released 2013. *P* value < 0.05 was regarded as statistically significant and set a priori.

## Results

A total of 1798 persons were included. Mean age was 75 (SD 9.1) years; 56% were men. Mean eGFR (mean from capa and Lmrev) was 64.8 ml/min/1.73 m^2^. Characteristics of the study population are shown in Table 2.

**Table 2:** Background characteristics for the study population

N = 1832^a^	n	Minimum	Maximum	Mean	SD
Age	1798	64	101	75	9.15
p-creatinine (µmol/l)	1574	41	409	86	29.4
p-cystatine C (mg/l)	1574	0.61	5.89	1.14	0.43
eGFR LMrev (ml/min/1.73 m^2^)	1574	10.1	107	61.5	15.1
eGFR capa (ml/min/1.73 m^2^)	1574	5.0	126	68.1	20.3
eGFR mean (ml/min/1.73 m^2^)	1574	9.5	103	64.8	17.0
Mini-mental test, sum	1602	5	30	26.7	2.98

	n	Proportion (%)
Male	1000/1798	56
CKD grade 1-2	1032/1574	65.6
CKD grade 3	487/1574	30.9
CKD grade 4-5	55/1574	3.5
Dementia diagnosis		
Yes	51/1712	3.0
No	1565/1712	91.4
Unsure	96/1712	5.6
Educational level		
Elementary school not completed	57/1782	3.2
Elementary school	859/1782	48.2
Secondary school	495/1782	27.8
≥ One year extra or university degree	371/1782	20.8
Place of living (i.e. proximity of care/pharmacies)		
In rural areas	59/1690	3.5
In a village	112/1690	6.6
In urban areas	245/1690	14.5
In a city	1274/1690	75.4
Use of other analgesics than NSAID	83/1798	4.6

^a^Date for examination was missing for 34 cases

NSAIDs were used on a daily basis or as needed by 105 persons (5.8%). The mean eGFR was 64.0 (31.1–100.1) in this group. Of those taking NSAIDs, 23 were prescribed the medicine [mean eGFR = 64.8 (39.8–95.3)] and 82 were buying it OTC [mean eGFR = 63.8 (31.1–100.1)]. A total of 42% of those buying NSAIDs over the counter showed an eGFR below 60 ml/min/1.73 m^2^. The most frequently used NSAID was ibuprofen (200–400 mg). Four persons reported the use of two different NSAIDs similarly. The regular use of other analgesics (paracetamol, opioids or drugs for neuropathic pain) as a marker for chronic pain condition was reported by 83 persons. Table 3 shows the use of NSAIDs among the study population and within the groups of different CKD-stages.

**Table 3:** The use of NSAIDs (regularly or as needed) within the groups of different CKD-stages

	NSAID use, n (%)	NSAID prescribed by physician, n (%)	NSAID use OTC, n (%)
Study population, total (N = 1798)	105 (5.8)	23 (1.3)	82 (4.6)
CKD grade (n = 1574)^a^			
1–2 (eGFR ≥ 60 ml/min/1.73 m^2^)	57 (3.6)	14 (0.9)	43 (2.7)
3 (eGFR 30–59 ml/min/1.73 m^2^)	39 (2.5)	8 (0.5)	31 (2.0)
4–5 (eGFR < 30 ml/min/1.73 m^2^)	0 (0)	0 (0)	0 (0)

^a^eGFR was missing for 224 people

The multiple linear regression showed that female sex (beta = 0.029; 95% CI = 0.005–0.052) was weakly but significantly associated with NSAID use (*p* = 0.018). This was also true when NSAID use was split into no use/OTC/prescribed (beta = 0.042; 95% CI = 0.012–0.073; *p* = 0.007). The other independent variables (age, level of education, access to care and score level of mini-mental test) did not contribute to the model in any of the analyses. The respective R^2^ values were 0.007 and 0.009.

## Discussion

This study on the general elderly population in southern Sweden shows that more than one out of 20 among the study population, which is representative of Sweden as a whole, use potentially hazardous anti-inflammatory medication. Even elderly people with moderate kidney function decline (CKD grade 3) use NSAIDs, which suggests that many are unaware of the risks associated with this medication. Age or education degree did not affect the use of NSAIDs. However, there was a small but significant association between female sex and NSAID use.

Among those who showed a kidney function decline to grade three (n = 487), 8.0% used NSAIDs. Most of them (31 out of 39) bought these medications OTC. This finding stresses the importance of asking patients about OTC use and of informing them about risks. This is even more important if the patient is receiving treatment with other medications that are dependent on kidney function for their elimination, i.e. metformin, digoxin and spironolactone [33]. Even occasional intake of NSAIDs might cause acute kidney failure [11] with an according risk of accumulation and even fatal outcome. This study, however, did not assess the contemporary use of medications other than analgesics.

In Sweden it is relatively easy to buy NSAIDs OTC and the majority of the NSAID users in the study bought the drug OTC. Broadly speaking, every supermarket offers these products. Paracetamol tablets, however, are since 2015 (but not during the study period) only sold at pharmacies, due to repeated intentional self-harm acts. It would therefore not be surprising if the average elderly person was of the belief that NSAIDs are less dangerous than paracetamol. Consequently, it is important to provide information that these are not risk-free medications. Nurses who give advice to patients with momentary pain from the musculoskeletal system should not suggest NSAID for elderly people. Likewise, the communication between physicians and staff who meet these patients at the pharmacies could be developed and expand [34]. Physicians must also avoid prescribing these drugs for patients at risk of kidney failure as well as gastrointestinal bleeding and heart failure. Physicians should instead choose drugs in accordance with the most recent guidelines. Primary care patients with gonarthrosis did not report any difference in knee pain severity between those taking paracetamol or diclofenac. Furthermore, the patients more frequently reported minor adverse events after taking diclofenac (64%) than paracetamol (46%) [35].

The regular use of other analgesics (paracetamol, opioids or drugs for neuropathic pain) as a marker for chronic pain condition was reported by 83 persons (4.6%). This was less than those using NSAIDs, which 105 elderly people (5.8%) did. This finding implies that NSAIDs might be used as a first choice drug, instead of analgesics recommended for elderly, especially paracetamol. This was seen previously in Germany, where ibuprofen was the most frequently used analgesic [36], while in Norway paracetamol use was twice as common as NSAID use [37].

The regression did not show an association between age and NSAID use. This is risky since it implies that the oldest, although they are most likely frailer and have lower kidney function, use these medications to the same extent as the younger elderly do. In the general Italian population, the older age groups even showed an increased risk of chronic NSAID use, according to a survey performed in 2002 [19]. Furthermore, in Germany in 2014–2015 a fifth of the nursing home patients, who often are the frailest and oldest, were prescribed NSAIDs, regardless of kidney function state [38]. In Sweden, physicians seem to be more careful with NSAID prescriptions to the frailest patients [5]. However, the problem with OTC use still remains. This implicates that there is a need for developing the role of community pharmacists in providing counselling to the patients regarding NSAID use.

In a previous Swedish study, lower education level has been shown to be associated with a higher risk of hazardous medication use [39]. In the present study, however, education level was not found to associate with NSAID use, and nor was cognition level (MMSE). The small but significant association between female sex and NSAID use was not surprising. The fact that women use more analgesics, both prescribed and OTC, has been shown previously [36, 37].

The medication lists that were collected contain data about prescription drugs as well as OTC drugs. This is a strength compared to studies with collected data from the Swedish Prescribed Drug Register [40]. Access was given to medical records in order to check medications that were prescribed. However, medication data refers to stated intake from prescriptions as well as OTC. Other strengths that should be pointed out are that home visits were performed in order to minimize selection bias of healthier individuals. The study design with urban and rural areas increases the generalization of our findings.

One weakness of the study is that it was not possible to decide if the study person used NSAIDs regularly or more seldom, when it was reported as “as needed”. However, even a single intake of NSAID carries a risk of causing acute kidney failure [11] and the risk of myocardial infarction was recently found to be greatest during short term daily use of an NSAID [8]. It is also not possible to guarantee that all occasionally used NSAIDs were reported. Furthermore, it was not known how many of the patients taking NSAIDs who had an increased risk for adverse effects of those drugs, such as recent gastrointestinal bleeding, cardiac failure or renal failure. Missing data were most frequently seen regarding blood samples (258/1832). However, we had no reason to believe that the kidney function was different in this group.

Future research should focus on interventions to decrease NSAID, targeting prescribers and personnel at community pharmacies as well as elderly in general. Such studies could have the potential to form the basis of future strategies for optimizing drug use among the elderly. Assessing the knowledge about NSAIDs among older people who buy these medications OTC at pharmacies could be helpful in customizing information directed at the general elderly population in Sweden. Even more, perhaps regulation of OTC dispensing to the elderly should be a priority?

## Conclusion

Almost six percent of the elderly in our study population were using NSAIDs, which are potentially hazardous medications for this population. The majority bought these medications  over the counter, without prescription. Among those buying NSAIDs over the counter, almost half showed an eGFR below 60 ml/min/1.73 m^2^, suggesting that many people are unaware of the risks associated with the use. Education level did not affect the use of nonsteroidal anti-inflammatory drugs, nor did age. The latter is risky since it implies that the oldest, although they most likely are frailer and have lower kidney function, use these medications to the same extent as the younger elderly do. Many pain patients choose these medications as their first choice, instead of other recommended analgesics. It is important to provide the information that these are not risk-free medications, especially to the general population but also to physicians.
